# 547. The Use of Autologous Skin Cell Suspension in Treating Adult Facial Burns: Single Institution Series

**DOI:** 10.1093/jbcr/irag033.244

**Published:** 2026-04-06

**Authors:** Natalie F DeWitte, Lori Rhodes, Danielle L Hashmi, Devon Bodeker

**Affiliations:** Temple Burn Center, Temple University Hospital, Philadelphia, PA; Temple University Hospital, Philadelphia, PA; Towerhealth, Reading Hospital, West Chester, PA; Temple University Hospital, Philadelphia, PA

## Abstract

**Introduction:**

Facial burns represent a challenging subset of burn injuries. The face is central to identity, communication, and sensory function. Management involves local wound care, infection prevention, and grafting. New technologies have aimed to accelerate burn wound healing. One such method produces autologous skin cell suspensions from a small split-thickness donor site, applied as a spray to burn wounds. It is approved for partial-thickness burns or with meshed autografts for full-thickness burns. While widely recognized in burn care, limited data exist on its use as a single therapy for facial burns.

**Methods:**

This study evaluated the utility of autologous skin cell suspension in adult facial burns. A retrospective chart review was performed at a single urban, ABA verified academic burn center from 03/2022 to 07/2025. Data collected included demographics, burn characteristics, operative conduct, complications, and media documenting healing progression and long-term outcomes.

**Results:**

Seventeen patients treated with autologous skin cell suspension as a primary dressing were included. The cohort was 65% male and 35% female, mean age 46 years. Mean total body surface area burned was 29%, with a mean hospital stay of 44 days. Injuries were primarily flame (82%), followed by chemical (13%) and scald (5%). All burns are classified as mixed depth as based on documentation and media review. No patient received concomitant skin grafting for areas of autologous skin cell suspension application as part of this review. Postoperative dressings included an absorbable synthetic wound dressing (65%) or non adherent, non absorbent, clear film dressing (35%). The most frequent complication was wound infection, as found in two patients (12%). Three patients (17%) required repeat treatment. Eight patients (47%) required ablative laser therapy for hypertrophic scarring of the face. Three patients (17%) underwent staged procedures with excision and allograft followed by re-excision and application of autologous skin cell suspension.

**Conclusions:**

This review suggests autologous skin cell suspension may have a role in adult facial burn management. Given the complexity of facial burns and the importance of optimizing function and cosmesis, donor-sparing approaches need further study. Although limited by small sample size and retrospective design, this series highlights the potential of autologous skin cell suspension as an adjunct in treating facial burns. Larger prospective studies are needed to clarify its impact on healing, long-term outcomes, and quality of life. Limitations include availability of follow up of more recent cases for need for ablative laser and equipment availability at the end of the study period.

**Applicability of Research to Practice:**

This study provides preliminary insight into the use of autologous skin cell suspension as a donor-sparing technique that may support favorable cosmetic and functional outcomes in facial burns.

**Funding for the study:**

N/A.

